# Catalytic Activity of Various Carbons during the Microwave-Initiated Deep Dehydrogenation of Hexadecane

**DOI:** 10.1021/jacsau.1c00326

**Published:** 2021-09-29

**Authors:** Xiangyu Jie, Jiale Wang, Sergio Gonzalez-Cortes, Benzhen Yao, Weisong Li, Yige Gao, Jonathan R. Dilworth, Tiancun Xiao, Peter P. Edwards

**Affiliations:** † Inorganic Chemistry Laboratory, Department of Chemistry, University of Oxford, South Parks Road, Oxford OX1 3QR, U.K.; ‡ Department of Materials, University of Oxford, Parks Road, Oxford, OX1 3PH, U.K.; § School of Chemical Engineering & Technology China University of Mining and Technology, Xuzhou, Jiangsu Province 221116, People’s Republic of China

**Keywords:** carbon materials, microwave-initiated catalysis, surface structure and catalysis, decarbonization, dehydrogenation, deactivation mechanism

## Abstract

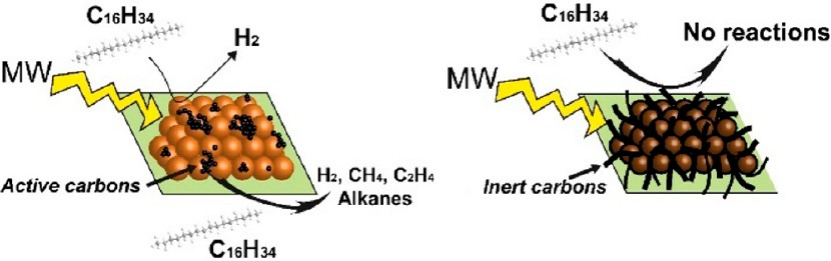

Carbon materials have been widely used as microwave susceptors in many chemical processes because they are highly effective at transforming incoming electromagnetic energy for local (hot spot) heating. This property raises the intriguing possibility of using the all-pervasive carbonaceous deposits in operating heterogeneous catalytic processes to augment the catalytic performance of microwave-initiated reactions. Here, the catalytic activities of a range of carbon materials, together with carbon residues produced from a “test” reaction—the dehydrogenation of hexadecane under microwave-initiated heterogeneous catalytic processes, have been investigated. Despite the excellent microwave absorption properties observed among these various carbons, only activated carbons and graphene nanoplatelets were found to be highly effective for the microwave-initiated dehydrogenation of hexadecane. During the dehydrogenation of hexadecane on a Fe/SiC catalyst, active carbon species were formed at the early stage of the reactions but were subsequently transformed into filamentous but catalytically inert carbons that ultimately deactivated the operating catalyst.

## Introduction

Carbon deposition on the surface of catalysts is a ubiquitous challenge for heterogeneous processes involving hydrocarbon reactants and products. It has been well-established that coke and other carbonaceous materials formed during these catalytic reactions will quickly deactivate catalysts by blocking catalyst pores and/or completely encapsulating the metal catalysts.^1,2^ This so-called “coking problem” hinders the development of many industrial processes. One such process gaining much attention involves the catalytic deep dehydrogenation of hydrocarbons (for example, methane) for producing pure hydrogen and solid carbon, a process that is particularly important as it proceeds without CO_2_ formation.^3−6^ Although extensive work and progress have been made in tackling the coke problem, considerable challenges remain.^7−9^

Microwave-initiated heating has recently become a useful tool in catalysis due to its fundamentally different operating mechanisms from conventional thermal methods.^10,11^ Microwave heating (also known as dielectric heating) occurs through the specific interactions between materials and the electric (or alternatively magnetic) field component of electromagnetic radiation.^12−14^ Thus, dipolar polarization effects, for example, occur with constituent polar molecules such as water etc., where the molecules attempt to align themselves under the alternating electric field. Similar effects arise from the presence of itinerant or conduction electrons in metallic catalyst particles. Such interfacial polarization is known as the Maxwell–Wagner polarization effect, which generates heat via charged particles generated by the accumulation of electrons at the boundaries or surfaces.^12−14^

Carbon materials have long been used as microwave receptor/acceptors for effective energy transfer because of their widely recognized highly efficient microwave absorption. Dielectric heating by high-frequency electromagnetic radiation occurs for carbon particles and causes them to rapidly heat up.^15^ However, despite the continually increasing interest in using microwaves in many areas of chemistry, the fundamental nature of the interaction of materials (catalysts) and chemicals (reactants) with microwaves remains unclear.^10,11^ Carbon materials clearly interact with microwaves to initiate the physical heating process, but there is still a lack of data on the potential ensuing catalytic activity of the developing carbon deposits on catalysts in their various forms. This is important because, if the processes from developing hydrocarbon decomposition or dehydrogenation reactions are themselves “microwave active”, this could potentially prolong the lifetime and possibly affect the very nature of the operating catalytic process itself. A deep understanding of precisely which types and forms of carbon can act as catalysts could open up new areas for utilizing carbon materials cooperatively as both microwave susceptors and catalysts.

Carbon-based catalysts have been used as microwave receptors to ensure the direct heating of nonpolar materials and studied for microwave-initiated catalytic reactions such as the decomposition of methane,^16,17^ carbon dioxide reforming,^18^ the pyrolysis of biomass and organic wastes, etc.^19,20^ The catalytic activity of carbons has also been studied in conventional thermal processes. Pioneering research by Muradov and colleagues^21,22^ reported that the disordered forms of carbon (e.g., activated carbon and carbon black) are more catalytically active than the ordered carbonaceous forms such as graphite and diamond powder. Moliner et al. also carried out a detailed study of the deactivation mechanism of carbon blacks for methane decomposition and proposed that the carbon crystallites formed during the reactions could create new active sites on the surface.^1^ Thus, carbon in its various forms clearly performs differently in its catalytic activities. Some types of carbon such as carbon black produced from the dehydrogenation of hydrocarbons are reported to be catalytically active, whereas graphitic carbons are not.^16,23,24^

We therefore set out to examine various carbon materials (having different geometries and surface structures) under microwave initiation for both their intrinsic and individual microwave absorption properties as well as their ensuing catalytic performance.^16,17,25,26^ For a model system,^27^ we have monitored the time dependence of carbon deposition/transformation on Fe/SiC catalysts through successive cycles of the microwave-initiated deep dehydrogenation of hexadecane.

## Results and Discussion

### Catalytic Activity of Different Carbons in Interaction with Microwaves

Carbon materials typically have different structures and origins,^16,28,29^ and therefore we selected the nine different kinds of carbon materials shown in Table 1 and tested their catalytic activities for the dehydrogenation of hexadecane under microwave irradiation, all attempted to be operating under—as close as possible—the same conditions.

**Table 1:** BET Surface and Porosity of Model Carbon Materials

model carbon material	BET surface area (m^2^/g)	pore size (nm)
activated charcoal	875	3.8
activated carbon (Norit)	864	3.8
graphene nanoplatelets	460	6.7
mesoporous carbon	734	17.4
glassy carbon	1.8	7.6
carbon black	65.1	16.5
CNFs	14.7	17.8
MWCNTs	255	20.5

**Figure 1:**
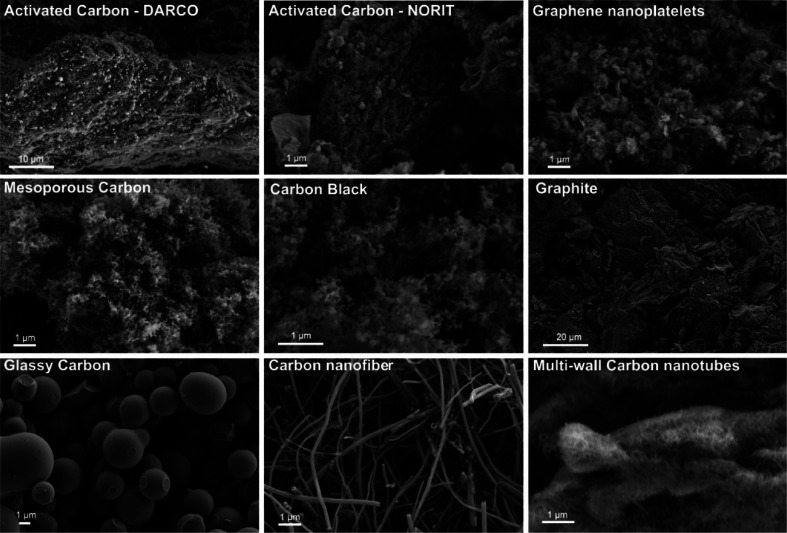
Scanning electron microscope (SEM) images of model carbon materials investigated in this work.

We show in Figure 1 the different morphologies and structures of the various selected carbons as characterized by SEM and a BET analysis (Table 1). Activated carbon (AC), carbon black (CB), and mesoporous carbon (MC) have a rough surface morphology with evident porosity. In contrast, glassy carbon, carbon nanofibers (CNFs), and multi-walled carbon nanotubes (MWCNTs) have very smooth surfaces and a filamentous structure is clearly detected for CNFs and MWCNTs. In addition, graphite and graphene nanoplatelets (GNPs) are primarily composed of layered carbon structures. In general, disordered carbons such as ACs and GNPs have very high surface areas, ranging from 460 to 875 m^2^ g^–1^ with a small average pore size of ca. 3.8–6.7 nm. The surface areas of other carbon materials including CB, glassy carbon, and CNFs are typically less than 80 m^2^ g^–1^. MWCNTs have a moderate surface area of 255 m^2^ g^–1^ with the largest pore size of 20.5 nm.

**Figure 2:**
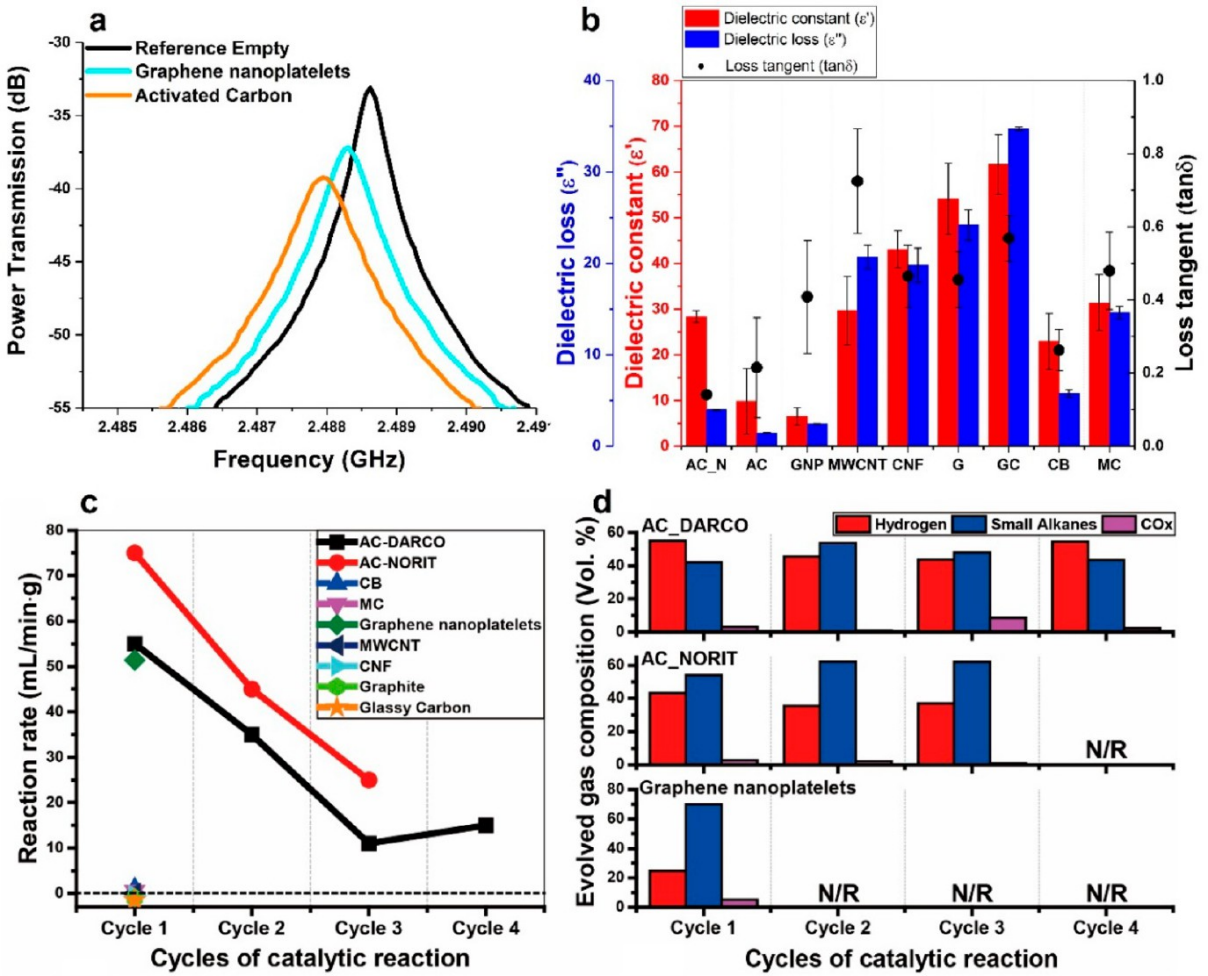
(a) Cavity perturbation measurements of selected carbon materials of representative captured microwave plots induced by activated carbon and graphene nanoplatelets. (b) Dielectric constant, dielectric loss, and loss tangent of nine different kinds of carbon materials; activated carbons (AC), carbon black (CB), mesoporous carbon (MC), graphene nanoplatelets (GNPs), multi-walled carbon nanotubes (MWCNTs), carbon nanofibers (CNFs), graphite (G), and glassy carbon (GC). (c) Hexadecane dehydrogenation over different carbon materials, with the reaction rate valued by the volume of gases collected per minute of 1 g of the catalyst. (d) Evolved gas composition in the exit gas. Between each successive cycle of the tests, an additional fresh 0.5 mL of hexadecane was added to the resulting residues.

We first measured the dielectric properties of these selected carbon materials by the microwave cavity perturbation method.^13,30,31^ The dielectric constant (ε′) of a material represents its ability to store electrical potential energy under microwave irradiation, while the dielectric loss (ε′′) quantifies the efficiency with which the absorbed energy is converted into heat. The loss tangent (tan δ) is introduced as ε′ divided by ε′′, indicating the ability to convert electromagnetic energy into heat energy at a given frequency and temperature.^27^ The method is based on the change in the frequency curve (Figure 2a) of the resonance and the *Q* factor of the cavity^32,33^ (details are given in Methods). The dielectric constant (ε′), dielectric loss (ε′′) and the loss tangent (tan δ) are then calculated based on the change in the resonant frequency Δf and bandwidth ΔBW from the measurements.

In Figure 2b we illustrate the dielectric properties measured at room temperature of all the selected carbon materials. The dielectric constant (ε′), dielectric loss (ε′′) and loss tangent (tan δ) of these different carbon materials are highly dependent on the type of carbon and the individual structures. Thus, carbons with ordered structures such as MWCNTs, CNFs, graphite, and glassy carbon have typically higher ε′, ε′′, and tan δ value in comparison to those of the disordered carbons of AC and GNPs.

Despite these various differences, all of the carbons presented an excellent ability to absorb microwaves and consequently heat up. We note also that the dielectric properties of the carbons are negligibly affected by their increasing temperature upon microwave irradiation (Figure S1).

In microwave heating, carbon materials rich in itinerant or conduction electrons—notably, delocalized π-electrons—will interact most effectively with microwaves and generate heat via Joule heating within the grain or arc generation at phase boundaries between the grains.^34^ Thus, importantly, key aspects of the heating mechanism and any catalytic reaction occur at the carbon surface and interfacial boundaries. Earlier important work by Compton and colleagues^35^ demonstrated in electrocatalysis the relationship between the surface structure of carbons and the electrochemical and chemical reactivity. They concluded that edge-plane sites and tube ends of graphitic carbon are the reactive sites and that much of the catalytic activity, electron transfer, and chemical reactivity of graphitic carbon is at surface defect sites, in particular edge-plane-like defect sites. We therefore follow this explanation and believe the precise catalytic properties of carbon materials under microwave irradiation depend strongly on the surface structure.

We then tested the catalytic performance of each model carbon material through the microwave-initiated catalytic dehydrogenation of hexadecane. Typically, carbon samples were blended with an aliquot of hexadecane in a weight ratio of 1:1. Then, the samples were exposed to 750 W microwaves for 10 min. Among these model carbon materials, only AC and GNPs show significant catalytic activity (Figure 2c). AC produced more hydrogen in comparison to GNPs, while more small hydrocarbons such as methane and ethylene were generated over GNPs. It should be noted also that the AC samples purchased from different companies gave different catalytic performances in terms of product distribution. More hydrogen was selectively produced over AC from DARCO while AC from NORIT gave more small hydrocarbons (Figure 2d). Moreover, AC and GNPs achieved temperatures of 500–600 °C, higher than those of other types of inactive carbon materials.

As a general observation, carbons such as AC and GNPs with high surface areas and small pores are highly catalytically active in the specific dehydrogenation process, whereas carbons with low surface areas and large pores such as CB and CNFs are catalytically inert under microwave initiation. However, we also note that some earlier works claimed that there was no apparent correlation between the surface area of carbons and their catalytic activity in methane decomposition (CMD) under conventional thermal conditions when carbons were used as catalysts.^1,21^ On the other hand, several papers^7,36,37^ have suggested the importance of the pore morphology (pore size, porosity, and tortuosity). Carbon materials with an improved pore size distribution such as mesoporous and hierarchical porous carbons gave higher and more stable thermal catalytic activity in CMD.^7,38^ In the present study under microwave initiation, we found the catalytic activities of different carbon materials are highly structure dependent, for which the surface roughness, surface area, and pore size could affect the operating carbon catalysts.

Notably, carbon thus plays two roles simultaneously in the reaction: first, it acts as a microwave receptor to efficiently convert microwave electromagnetic energy into heat, and second, the ensuing catalytic reactions at the surface/boundaries take place upon reaching the necessary temperature. Since both the Joule heating and the catalytically reactive sites occur at surface defect sites and phase boundaries of carbons,^34,35^ it is reasonable that disordered carbon materials are more active than carbons with ordered graphitic structures.

In addition, we have carried out successive cycle tests involving the addition of fresh hexadecane between each cycle of the experiment (Figure 2c,d) which shows that the AC and GNPs (active carbon species) were deactivated within the accumulation of carbon residues throughout the reactions. This suggests that carbons produced and deposited during the dehydrogenation reactions are catalytically inert upon microwave irradiation and could subsequently deactivate the operating carbon materials by covering the active sites. This also led us to believe that much of the catalytic activity appearing in carbon materials is from the surface defect sites and that the continuous carbon formation/deposition will cover the reactive sites and could subsequently lead to the formation of other types of carbons during the reactions.

### Evolving Nature of Carbon Formation throughout the Microwave-Initiated Dehydrogenation of Hexadecane on Fe/SiC Catalysts

**Figure 3:**
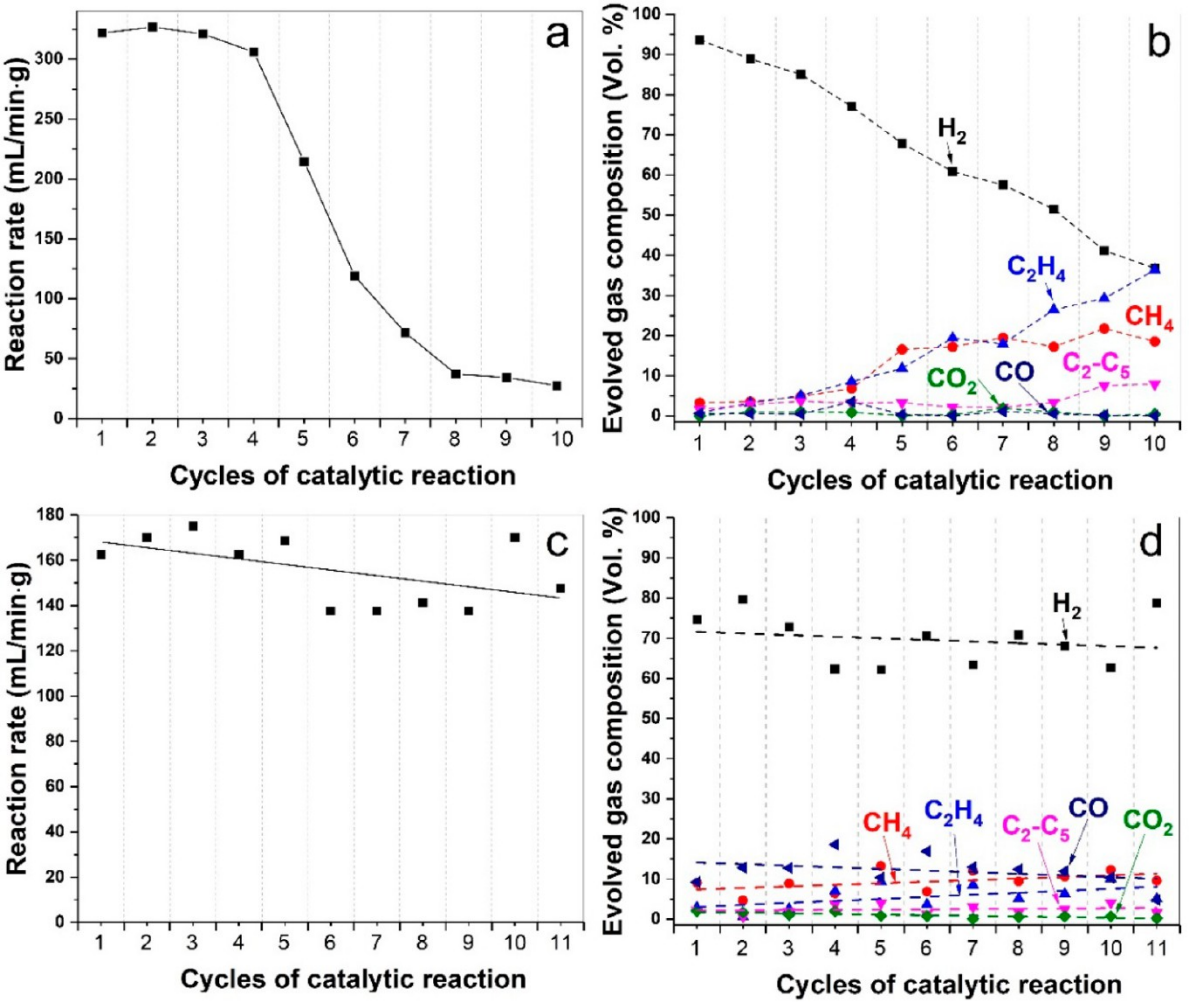
(a, b) Successive cycles of hexadecane dehydrogenation over 5 wt % Fe/SiC catalyst. The sample was refueled with 0.5 mL of fresh hexadecane between each cycle, and every cycle of the tests was run for 10 min under 750 W irradiation. (a) C_16_H_34_ dehydrogenation rate. (b) Evolved gas composition in the exit gas. (c, d) Successive cycles of regeneration tests of spent Fe/SiC samples. The spent catalysts were calcined at 550 °C in air in order to remove deposited carbon residues; then the obtained Fe_2_O_3_/SiC samples, without further hydroreduction, were tested for hexadecane dehydrogenation under the same conditions. (c) C_16_H_34_ dehydrogenation rate. (d) Evolved gas composition in the exit gas.

The nature and amount of carbon production/deposition and the ensuing catalytic activity were carefully monitored via successive cycles of the microwave-initiated hexadecane dehydrogenation on Fe/SiC catalysts. Despite the accumulating carbon deposition, the Fe/SiC catalyst continued to function for several catalytic cycles through successive additions of fresh aliquots of substrate to the reactor system; a typical sequence is shown in Figure 3a,b. Throughout the successive cycles of catalytic reactions, the catalytic activity of the Fe/SiC catalyst gradually decreased while the distribution of the evolved gaseous products changed. The reaction rate of the Fe/SiC catalyst started to decrease at the third cycle and remained at a low quasi-steady level after eight cycles of experiments (Figure 3a). The evolved gas selectivity for hydrogen gradually decreased, while the corresponding methane and ethylene concentrations increased (Figure 3b). We attribute these to the formation of carbons that could deactivate Fe catalysts and the participation of some active carbons in the catalytic dehydrogenation of hexadecane, resulting in the varied product distributions.

To further demonstrate the effect of depositing carbons, the fully spent catalyst after 10 successive cycles of experiments was recovered by aerial combustion at a temperature of 550 °C in order to remove all the carbon residues (Figure 3c,d). The resulting Fe_2_O_3_/SiC samples, without further hydroreduction, were then tested for dehydrogenation of hexadecane under microwave irradiation. The Fe_2_O_3_/SiC catalyst is itself a good microwave absorber, and Fe_2_O_3_ nanoparticles on the SiC surface can also catalyze the dehydrogenation reactions under microwave irradiation.^11^ However, a noticeably increased amount of CO_*x*_ was produced during the reactions with Fe_2_O_3_ in comparison with the Fe/SiC catalyst. We repeated the regeneration process by 10 successive cycles (Figure 3d), in which the spent samples were calcined and then refueled with fresh hexadecane between each cycle of tests and then exposed to microwave irradiation for dehydrogenation reactions.

As a general observation, the “recovered” samples were fully functional under subsequent microwave initiation, and both the reaction rate and the product distribution remained approximately constant throughout the 10 successive cycles of regeneration as a result of removing carbon between each cycle.

In comparison to the unregenerated catalysts (Figure 3b)—that is without removal of carbon residues—a noticeable increase in C_1_–C_4_ was observed throughout the 10 successive cycles of tests. This is despite the fact that the catalytically reactive Fe is presumably still available and potentially dominant in the early cycles of our tests (as was evident in our microscopy studies). The measured increase in olefin formation is attributed to the catalytic activity of at least some of the deposited carbons and carbides.

This illustrates that the advancing carbon deposited on the Fe/SiC catalyst surface in the early stage of dehydrogenation reactions is indeed catalytically active under microwave irradiation, hence preferably cleaving C–C bonds and producing methane, ethylene, etc.

If we return to the differences observed between the controlled cycles of experiment with, and without, removing deposited carbon, the data suggest the inert carbons formed can finally deactivate both the virgin metal catalysts and active carbon that was formed at an early stage of our tests by covering the active sites. Furthermore, the data suggest that the individual, characteristic nature of the surface structure of carbons is important in relation to exhibiting catalytic activity under microwave irradiation. As both the catalytic activity and electron transfer occur at the surface defect sites of carbons,^35^ the continual production and deposition of carbons from dehydrogenation will obscure these defect sites and subsequently convert any catalytically active carbon species to inert carbons under continuous microwave irradiation.

### Pre- and Postreaction Analysis on Carbon Residues in Comparison with Different Model Carbons

**Figure 4:**
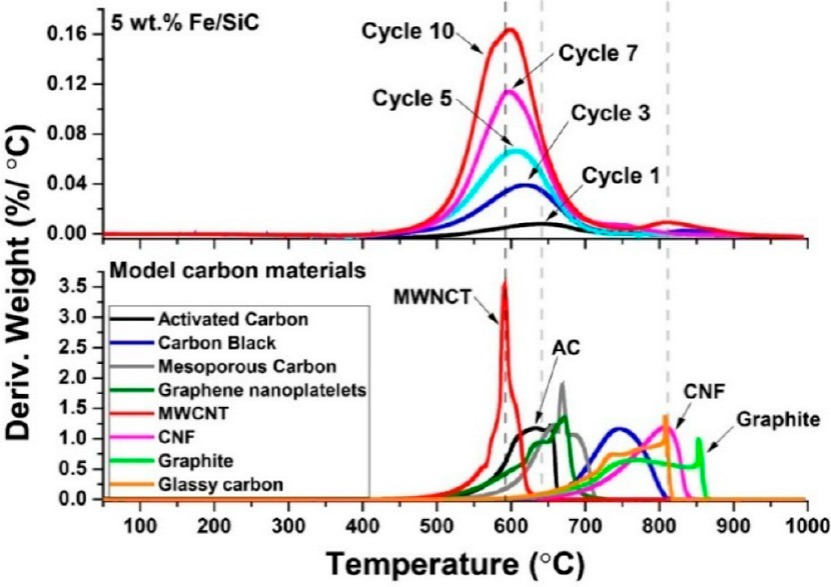
Derivative plots of temperature-programmed oxidation (TPO) of the spent Fe/SiC sample in comparison to the different carbon materials.

Through successive cycles of dehydrogenation reactions, carbon residues were constantly accumulated on Fe/SiC catalysts, as confirmed by our thermogravimetric analysis (TGA) (Figure S2). The oxidation temperatures of the resulting carbons from each cycle are similar at about 600 °C (Table S1). In Figure 4, we present the temperature-programmed oxidation (TPO) of spent Fe/SiC catalysts (with carbon depositions) in comparison with different carbon materials. The carbon produced at the early cycles has an oxidation temperature similar to that of AC at 650 °C, while after 10 cycles of reactions, the oxidation temperature of carbon residues in the spent catalysts shifted to about 590 °C, corresponding to the multi-walled carbon nanotubes (MWCNTs). A secondary peak at ca. 810 °C ascribed to the formation of carbon nanofibers (CNFs) and/or graphite was also observed.^39^

This once again clearly demonstrates that the carbon formed at early cycles of the tests are disordered active carbons (e.g., AC, GNPs), being rich in surface defect sites and highly catalytically active. In stark contrast, ordered carbons (e.g., MWCNTs, CNFs, and graphite), produced at later cycles, are catalytically inert and subsequently deactivate the catalyst by covering the reactive sites on both the iron and the carbon material.^7,40^

**Figure 5:**
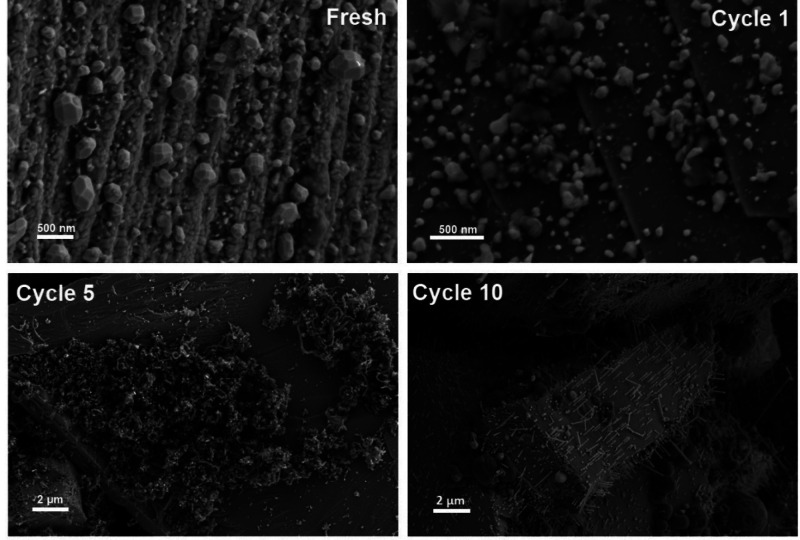
Scanning electron microscope (SEM) images of Fe/SiC samples through successive cycles of hexadecane dehydrogenation under microwave irradiation.

The changes in morphologies of carbon residues on the Fe catalyst surface are also observed in our scanning electron microscope (SEM) and transmission electron microscope (TEM) studies. The SEM images (Figure 5) show the evolving nature of Fe particles and accruing carbon formation on the spent Fe/SiC catalyst surface throughout the cycles of microwave-initiated dehydrogenation reactions. The deposited carbons were produced and then transformed from disordered carbon (highly catalytically active) clusters into the filamentous carbons (inert) as a result of more and more carbon being produced and deposited on the Fe catalyst surface under microwave irradiation.

Importantly, it can be seen that the reactive Fe catalysts were still very much in evidence, and indeed functioning catalytically, after cycle 1 and very few fragments of carbon were detected (Figure 5, cycle 1). Thus, no noticeable change in catalytic activity was found on Fe/SiC catalyst over the first three cycles of dehydrogenation reactions, while the evolved H_2_ composition monotonically decreased (Figure 3a,b).

Then, with time, when more carbon was deposited and accumulated on the Fe catalyst surface (after 5 cycles), the growth of many carbon clusters can be seen and filamentous carbons started to appear in these clusters. Interestingly, after 10 cycles of tests, these large clusters of carbon residues had all but “disappeared”; instead, the characteristic needlelike fibers of filamentary carbon were observed on the SiC surface.

**Figure 6:**
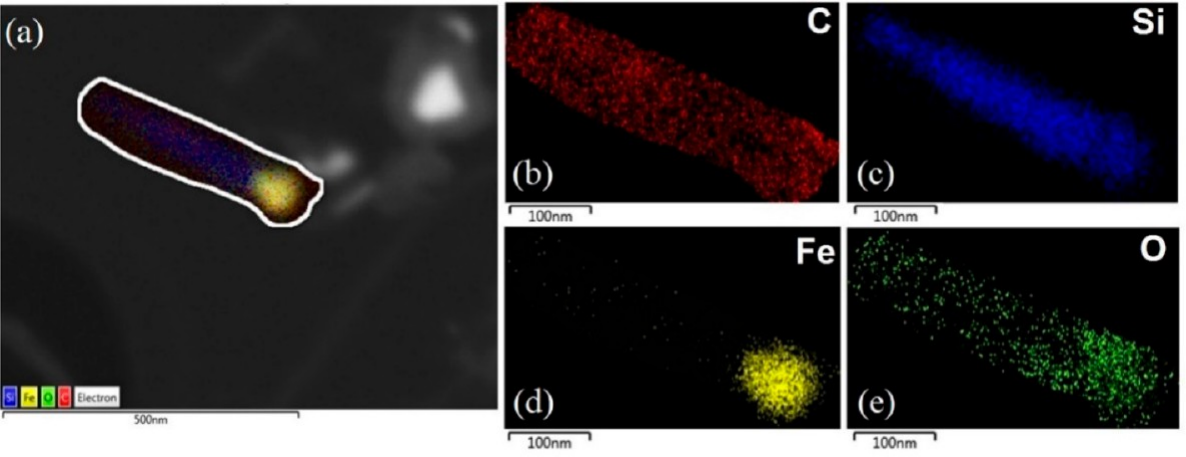
EDS mapping of needlelike fibers observed on the surface of SiC: (a) backscattered electron morphology; (b) C map; (c) Si map; (d) Fe map; (e) O map.

In Figure 6 we show the energy-dispersive X-ray spectroscopy (EDS) mapping of needlelike fibers observed on the surface of SiC after 10 cycles. The fibers have a core–shell-like structure. The top head consisted mainly of Fe, and Si was detected in the core. O and C were observed to be dispersed throughout the filaments, likely due to the presence of carbonyl, carboxylate, or hydroxyl groups, and C formed the shell of the fibers. Like the formation of carbon whiskers, it was assumed that this core–shell structure was formed by Si and C diffusion. Both C and Si diffused and dissolved into the Fe nanoparticles due to the temperature and concentration gradient between Fe nanoparticles and the SiC support.^41−43^ We attribute this to the localized high electric field and superheating caused by microwave-initiated effects that occurred at the surface and boundaries of the Fe nanoparticles and SiC support.^11,44^ Moreover, this effect will increase further by orders of magnitude due to the high depolarization factor of the nanostructures when the deposition of highly microwave absorbing carbon takes place.^11,45^ We note also that nonequilibrium plasma could be potentially generated in the interface of carbon residues, Fe nanoparticles, and SiC support by their close contact/proximity in the interaction with microwave irradiation.^46^ Thus, an enhancement in the average electric field strength in the boundaries of particles increases the probability of highly reactive collisions with active species in the plasma volume.

**Figure 7:**
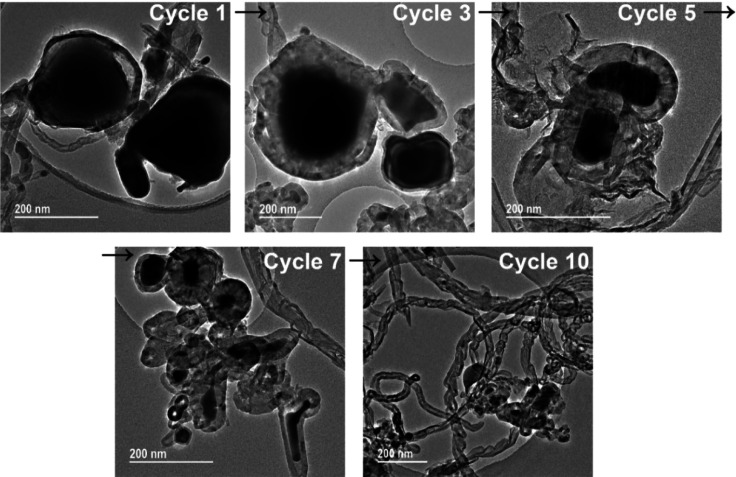
Transmission electron microscopy (TEM) of 5 wt % Fe/SiC catalyst throughout successive cycles of dehydrogenation of hexadecane under microwave irradiation.

Further studies on spent Fe/SiC samples by transmission electron microscopy (TEM, Figure 7) present the evolving changes in carbon formation on the surface of Fe catalyst particles that is evident in the SEM results. Very few carbons were observed on the Fe particles after cycle 1, and therefore, many fresh Fe active sites remained exposed and hence are catalytically active with newly added reactants. Following the continued carbon formation, more carbon was deposited and accumulated on the surface of the Fe catalyst particles. These carbons are primarily amorphous/disordered after 7 cycles of tests; filamentous carbons, MWCNTs, etc. were present and subsequently the MWCNTs were dominant after 10 cycles.

During the early cycles, carbons precipitate on the Fe catalyst surface and diffuse into the Fe to form Fe_3_C (as has been shown in our XRD studies, Figure S3). These carbons are primarily disordered carbons that are therefore highly active catalytically. As reported by Zhou et al.,^47,48^ the formation of Fe_3_C plays an important role in producing CNTs. Thus, with continuing carbon formation and precipitation in the later cycles of tests, carbons crystallize in the form of a cylindrical network and finally grow into catalytically inert tubular carbonaceous structures.

**Figure 8:**
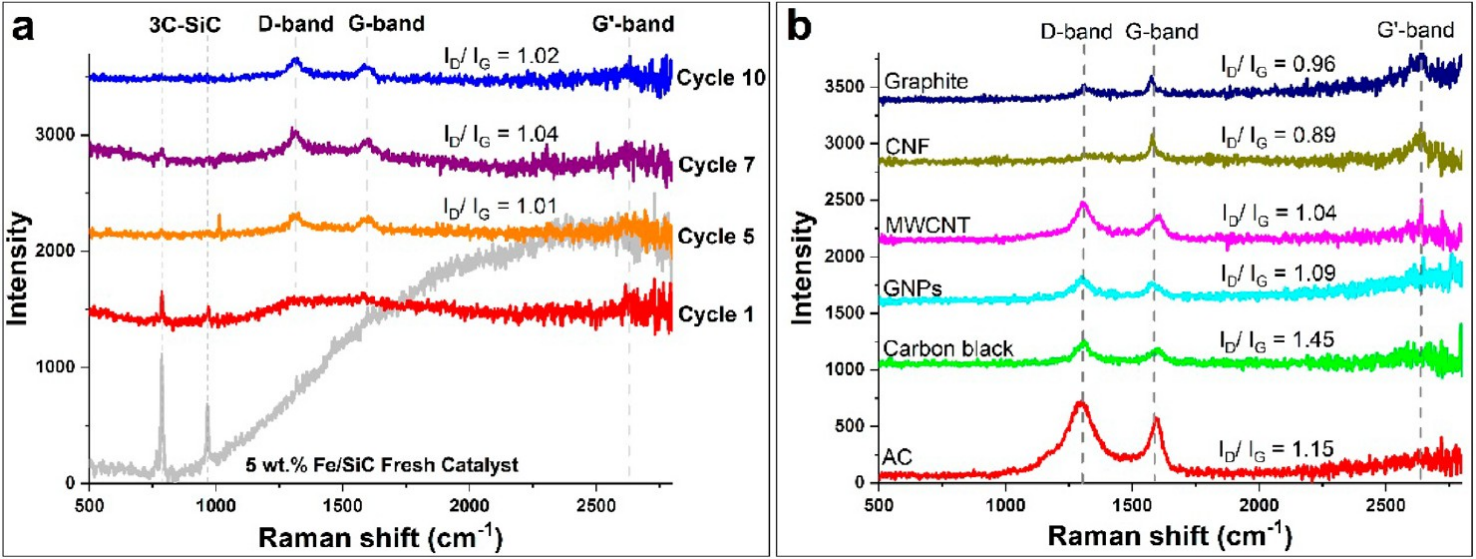
Raman spectra: (a) Sample of Fe/SiC through 10 successive cycles of catalytic reactions; (b) model carbon materials.

Raman spectroscopy and X-ray diffraction (XRD) studies also revealed the evolution of the particular carbon type. In Figure 8, the peaks observed at 1598 and 1313 cm^–1^ in the Raman spectrum correspond to the G and D bands, respectively. The intensity ratio of the D band to the G band (*I*_D_/*I*_G_) was then utilized to identify carbon possessing an aromatic ring structure. Throughout successive cycles of tests, the *I*_D_/*I*_G_ value of spent samples ranged from 0.97 to 1.04 (Figure 8a and Table S2) and higher *I*_D_/*I*_G_ values of about 1.02 were obtained at later cycles of tests which are close to the *I*_D_/*I*_G_ value of MWCNTs as observed in Figure 8b.

**Figure 9:**
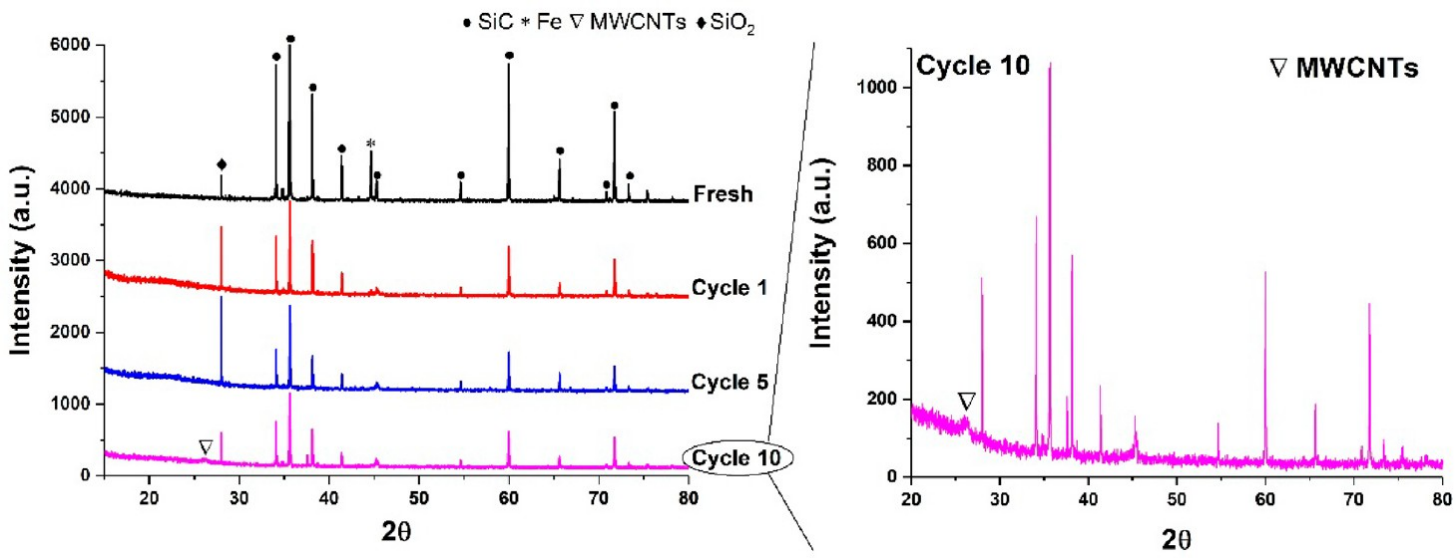
XRD patterns of a 5 wt % Fe/SiC sample throughout the successive cycles of dehydrogenation reactions under microwave irradiation.

XRD studies (Figure 9) on spent samples showed the changes in Fe catalyst particles and the formation of MWCNTs. The diffraction peak of metallic iron at the angle (2θ) of 44.8° was detected in the fresh Fe/SiC catalyst before the microwave treatment. After dehydrogenation reactions, the presence of Fe_3_C was detected (Figure S3). Moreover, an increasing intensity of the reflections was observed at 2θ = 28°, corresponding to amorphous and crystalline silicon, which is consistent with our EDS analysis on the spent samples. In comparison to the XRD patterns (Figure S3) of the nine kinds of model carbon materials, a very broad and low intensity peak detected at about 26° in the sample of cycle 10 is ascribed to MWCNTs.

**Figure 10:**
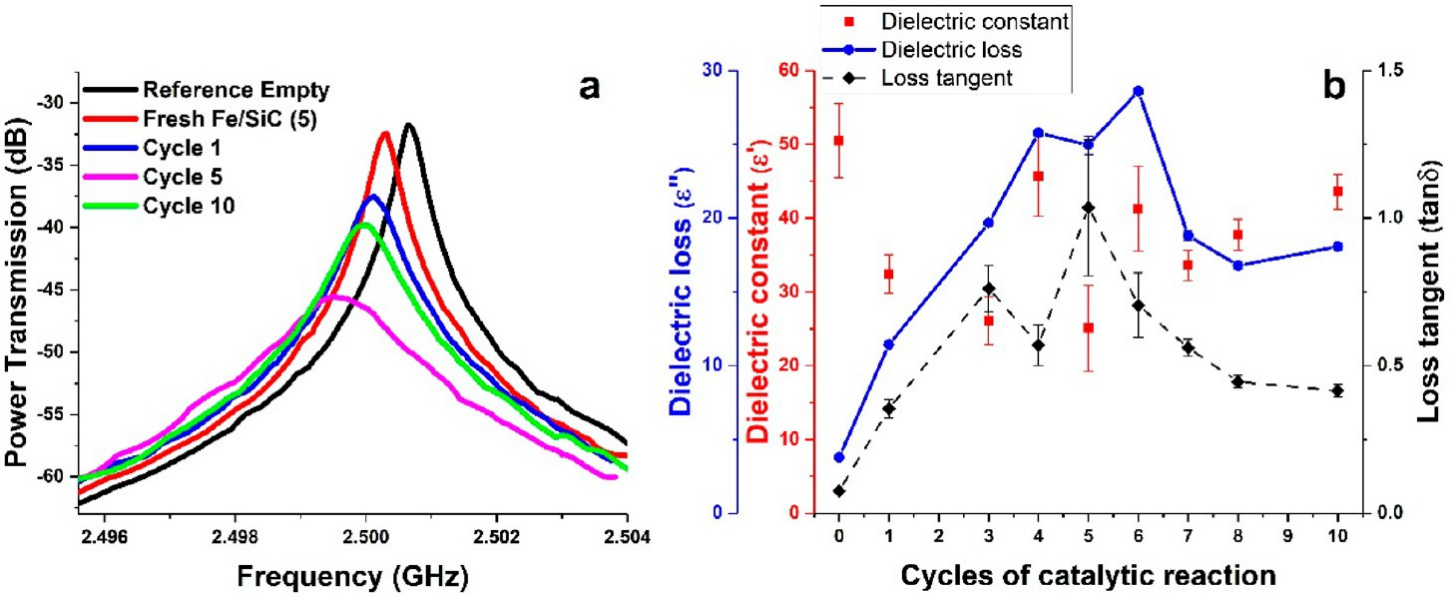
(a) Representative captured microwave plots induced by 5 wt % Fe/SiC samples through successive cycles of catalytic reactions under microwave irradiation. (b) Dielectric constant, dielectric loss, and loss tangent of 5 wt % Fe/SiC samples as a function of cycle numbers. The results shown were measured at room temperature.

As a result of carbon formation and transformation, the changes in the dielectric properties of the spent Fe/SiC catalyst were monitored using the microwave cavity perturbation method throughout successive cycles of microwave-initiated hexadecane dehydrogenation. We have previously shown that a microwave cavity perturbation analysis is an effective method to examine the cokes formed over zeolite catalysts.^45^ This technique can offer a rapid and nonintrusive measurement of catalytically coked catalysts on the basis of the detection of the changes in their dielectric properties.^13,30,31^ The change in the frequency curve (Figure 10a) of resonant frequency Δ*f* and bandwidth ΔBW were captured from the measurement, and the change in dielectric properties was then calculated.

In Figure 10b we display the evolving changes of dielectric properties of spent samples throughout the 10 successive cycles of dehydrogenation reactions. The dielectric constant of these spent samples was found to be between 25 and 50. In contrast, the dielectric loss and loss tangent of spent samples changed significantly. This can be ascribed to the evolved carbon deposition and transformation, since carbon materials effectively respond to the inducing microwaves. Typically, three steps of noticeable changes of dielectric loss and loss tangent of samples were detected during the reactions. From cycle 1 to cycle 3, the dielectric loss and loss tangent increased 6 and 10 times, respectively, which was attributed to the deposition of carbons and the resulting carbon diffusion into Fe particles to form Fe_3_C. Then, both the dielectric loss and loss tangent fluctuated at a certain range between cycle 4 and cycle 6, with the continuous deposition of carbon residues. The decrease in these dielectric values at later cycles of the tests suggests the formation/transformation of active carbon species that were produced at the early cycles into other forms of inert carbons such as graphite, MWCNTs, etc.

We note that a detailed interpretation of this complex and continually evolving (with time) catalytic system, involving a microwave-induced electric field, is indeed challenging to understand in detail. Thus, the change in dielectric properties of spent samples will be dependent on both the formation of iron carbides and deposition of carbons. As has been demonstrated in our post-reaction characterization of the evolving changes in the Fe catalyst, we believe that the formation of Fe_3_C occurs at the very early cycles of the tests. The XRD patterns (Figure 9 and Figure S3) of spent samples show that the diffraction peaks of Fe were dramatically decreased after cycle 1 and, instead, the presence of Fe_3_C was detected. Therefore, the change in dielectric properties of spent samples throughout the 10 successive cycles of tests is more likely due to the evolving changes in the carbons, as carbon materials effectively responded to microwaves and were sensitive to the cavity perturbation method.^45^

### Evolving Carbon Deposition/Transformation in Relation to Its Catalytic Activity under Microwave-Initiated Catalysis

The fundamental difference in using microwaves versus thermal heating to initiate carbon catalysts for catalytic reactions is that developing carbon deposits can play two roles: energy harvesting and transfer of incoming microwaves and also catalytic activity. Therefore, if the deposited carbons during the dehydrogenation of hydrocarbons are catalytically active, so, importantly, that could mitigate any presumed loss of activity in the developing carbon deposition process.

In our process, carbons produced at the early stage of the reactions from the dehydrogenation of hexadecane are clearly catalytically active. This is evident by the changes in product distribution, as has been described earlier (Figure 3). Following further carbon production, the carbon residues could then diffuse into the Fe catalyst (in the case of using the Fe/SiC catalyst) to form Fe_3_C. Previous work by Zhou et al. studied CMD mechanism models over Fe catalysts,^47^ where three steps were assumed to occur during the reaction: CH_4_ activation–decomposition on the Fe^0^ surface to produce H_2_ and amorphous carbon, formation of Fe_3_C, and diffusion of carbon into Fe^0^ to form Fe_3_C and an amorphous carbon transformation to graphitic carbon.^47^ Those authors advanced the important role of Fe_3_C in terms of producing carbon nanotubes (CNTs). We therefore believe—as has been demonstrated by our SEM (Figure 5), TEM (Figure 7), and cavity perturbation measurement (Figure 10) studies—that, following the formation of Fe_3_C at an early stage of the test, the active sites on carbons would be subsequently covered by inert carbons and the structure of carbon therefore varies. Finally, the formation of inert filamentary carbons will deactivate the catalysts.

**Figure 11:**
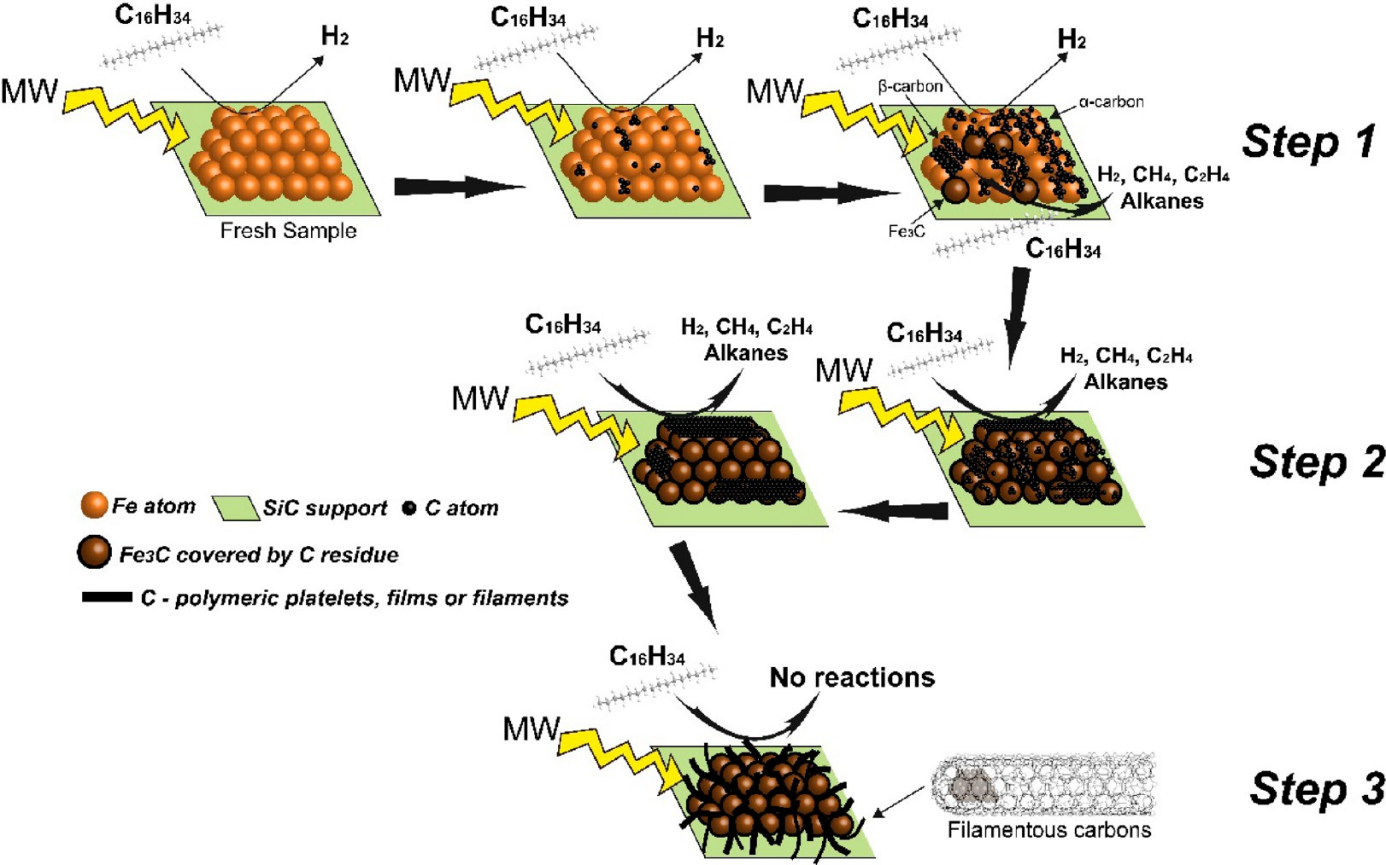
Representative diagram of carbon deposition and transformation through the microwave-initiated dehydrogenation of hexadecane over Fe/SiC catalysts.

In Figure 11 we have attempted to depict the carbon formation/transformation on an iron catalyst particle surface during the microwave-initiated dehydrogenation reaction. Thus, at the early stage of the test, Fe catalysts remain active at an initial level in spite of some carbons being formed. The Fe nanoparticles thus remain reactive at this stage and remain dominant in the catalytic reactions.

In the first step (Figure 10d), the C atom from C–H bond dissociation is chemisorbed strongly as a monolayer or physically adsorbed in multilayers on the Fe surface to form C_α_, where C_α_ indicates an adsorbed atomic carbon or surface carbide (eq 1).^2^ These C_α_ species should be disordered and have a large number of surface defect sites, so that are catalytically active under microwave irradiation. Following the continuous formation and deposition of C atoms, C_α_ can either react with newly precipitated C atoms to form C_β_ (eq 2), a polymeric carbon film, or penetrate into Fe to form Fe_3_C (eq 3). Moreover, C atoms could also be dissolved in Fe as vermicular carbon.^2^ Both α-carbon and β-carbon formed at the early cycles is considered catalytically active under microwave irradiation.
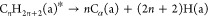
1
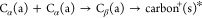
2
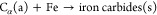
3

At the second step, following the accumulation of continuous carbon deposition, it results in saturation of Fe_3_C and the majority of the Fe active sites are covered by the carbons. From this point, the α-carbon (C_α_) is catalytically active and would be dominant in the reactions.^47^ This is reflected in the decreased reaction rates and the changed product distribution of the catalysts observed between cycle 4 and cycle 7 (Figure 3). Finally, these active carbons are gradually deactivated as a result of the active sites or the surface defect sites being covered by inert C. Hence, the active amorphous α-carbon and β-carbon that formed at the early cycles are converted/transformed into inert polymeric platelets, films, or filaments (eq 4). Consequently, the Fe and Fe_3_C particles are encapsulated/covered by the inert polymeric carbons and thereby the catalysts are deactivated.
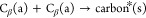
4In eqs 1–4 *(a) and (s) refer to adsorbed and solid states, respectively, carbon^+^ refers to amorphous and graphitic carbons, and carbon* refers to polymeric, graphitic platelets, films, or filaments.

## Conclusion

Carbon, in its various forms, shows highly different catalytic activities under microwave-initiated catalytic processes despite its overall excellent microwave absorption characteristics. We have demonstrated in this study that the surface structure of carbons is highly important and decisive for its catalytic activity for microwave-initiated catalysis. Among the nine different types of carbons tested for microwave-initiated hexadecane dehydrogenation, only AC and GNPs are catalytically active and other types of carbon are catalytically inert but can efficiently be heated under microwave irradiation.

A further investigation of the carbons produced from the dehydrogenation of hexadecane has illustrated the evolving nature of the carbon species in terms of their structure and catalytic activity. The carbons formed at an early stage of the successive cycles of tests remain catalytically active under microwave irradiation. However, these carbons ultimately transition into inert carbon materials, notably graphite, filamentary carbons, etc.; the greater amount of carbon deposited throughout the reactions subsequently encapsulates the active metal catalysts and covers the active sites on the active carbon compounds, thereby deactivating the catalysts.

With the increasing interest in microwave-initiated catalysis, we believe that the catalytic activity of different types of carbons under microwave irradiation is important, where the carbon is acknowledged and utilized as an effective microwave absorber. To be able to control the types of carbon formed during the reactions opens up the possibility of prolonging the lifetime of catalysts under microwave irradiation used for dehydrogenation reactions. Further work is certainly needed to formulate a sophisticated procedure to control the formation of specific types of active carbons. However, we believe this study provides some guidance for the design and use of carbon-based materials for microwave-initiated catalysis. On the basis of their unique origin, structure, and catalytic activity, different carbon-based catalyst systems can therefore be designed for important reactions through microwave-initiated catalysis.

## Methods

### Preparation of Catalysts

All of the carbon materials were used as purchased without further purification. Activated carbon, carbon nanofibers, and carbon nanotubes (multi-walled) were purchased from Sigma-Aldrich, and carbon black was purchased from Alfa Aesar.

The iron catalyst supported by silicon carbide was prepared by an incipient wetness impregnation method. The iron nitrate Fe(NO_3_)_3_·9H_2_O (iron(III) nitrate nonahydrate, Sigma-Aldrich) was used as the metal precursor, and SiC (silicon carbide, Fisher Scientific) was used as the supporting material. A certain amount of iron nitrate was dissolved in distilled water to prepare an aqueous solution, the concentration of which was calculated to produce a desired Fe loading (in this study, a 5 wt % Fe loading was applied). SiC powder was then added to the solution and mixture stirred at 150 °C for 3 h. The formed slurry was then moved into the drying oven and left overnight. A mortar was used to grind the dried bulk into a powder, which was calcined in a furnace at 350 °C for 3 h. Finally, the active Fe/SiC catalyst was obtained by reducing in 10% H_2_/Ar gases at 800 °C for 6 h.^27,39^

### Apparatus and Method Used for Catalytic Activity Evaluation

In accordance with a previous study, the process of dehydrogenation of hexadecane over the Fe/SiC catalyst was chosen as a prototype for the study of carbons under microwave irradiation. The experiments were conducted for successive cycles of tests using a purpose-built microwave cavity and a control system (the details of the microwave reactor were reported in our previous publications).^27,29^ A quartz tube (inner diameter 6 mm, outer diameter 9 mm) was filled with about 1.13 cm^3^ of the catalyst at a height of 4 cm , to allow the sample to be fully exposed to the axially polarized (TM_010_) electric fields. A 0.5 mL portion of hexadecane was then injected into the tube at each cycle of the tests. The sample was held for 5–10 min in order to get a full dispersion of the hexadecane in the catalyst bed. All experiments were conducted at atmospheric pressure, and the samples were purged with an Ar flow rate of 1.67 mL s^–1^ for a period of 15 min before the start of microwave activation. Typically, the sample was irradiated with microwaves for 10 min at 750 W in each cycle. A fresh 0.5 mL of hexadecane was refueled between each cycle of the tests. The generated gases were collected and analyzed by gas chromatography (GC) using a PerkinElmer Clarus 580 GC instrument. The catalytic performance of different carbon samples was tested following the sample methods.

All references to the catalytic activity of catalysts relate to the gas generation rate per unit of catalyst weight (in mL/(min g)). The selectivity to different gaseous products was described in terms of the volume percent of the product composition in the exit gases.

### Pre- and Post-reaction Analysis

The samples were carefully characterized before and after experiments by powder X-ray diffraction (XRD, BRUKER D8 ADVANCE), thermogravimetric analysis (TGA, TA Instruments, SDT Q-600), laser-Raman spectroscopy (PerkinElmer RamanStationTM 400F spectrometer), and scanning electron microscopy and energy-dispersive X-ray spectroscopy (SEM-EDS, ZEISS EVO).

The morphologies of the produced carbon residues were also examined by transmission electron microscopy (TEM) using a JEM-3000F microscope (300 kV). The sample powder was dispersed in ethanol in an ultrasonic bath for 15 min. The solution was then drop-cast onto a 300 mesh copper TEM holey carbon grid on a filter paper and allowed to evaporate. Scale bars of all the TEM images were calibrated using an oriented gold crystal grid.

In addition, the changes in the dielectric properties of Fe/SiC catalysts and different carbons throughout the successive cycles of dehydrogenation tests were measured via microwave cavity perturbation measurements (transverse magnetic TM_010_ mode; more details are available in our previous publication^27^). Measuring dielectric properties of a material using the perturbation technique is based on the change in the frequency curve of the resonance and the *Q* factor of the cavity.^32,33^ The dielectric constant (ε′), dielectric loss (ε′′), and loss tangent (tan δ) were then calculated on the basis of the change in the resonant frequency Δ*f* and bandwidth ΔBW from the measurements by eqs 5–7^13,30,31^
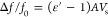
5
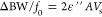
6
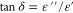
7where *A* is a constant determined by the size and geometry of the cavity and *V*_s_ is the effective volume of the sample in the cavity. In this work, the value of *A* is approximately 7.114 × 10^–3^ and that of *V*_s_ is about 1.272 × 10^–3^ cm^3^.

## Supporting Information Available

More details of pre- and post-reaction characterization, cavity perturbation measurements of activated carbon and graphene nanoplatelets at different temperatures, thermogravimetric analysis of cyclic experiments, XRD patterns of model carbon materials and Fe/SiC samples, and Raman spectra analysis of model carbon materials and Fe/SiC samples (PDF). The research materials supporting this publication are contained within the paper and its associated Supplementary Information. All other relevant data are available from the corresponding author upon reasonable request.
