# Supplementary material for: Catalytic Activity of Various Carbons during the Microwave-Initiated Deep Dehydrogenation of Hexadecane
Source: JACS Au. 2021 Sep 29;1(11):2021–32. doi: 10.1021/jacsau.1c00326 (PMC8611660; doi:10.1021/jacsau.1c00326)
Supplement: Supplementary file 1 — au1c00326_si_001.pdf [file au1c00326_si_001.pdf]

Supplementary Information for

Catalytic activity of various carbons during the microwave –initiated deep dehydrogenation of hexadecane.

Xiangyu Jie[1*], Jiale Wang[2], Sergio Gonzalez-Cortes[1], Benzhen Yao[1], Weisong Li[1,3], Yige Gao[1], Jonathan R. Dilworth[1], Tiancun Xiao[1*] and Peter P. Edwards[1*]

1. Inorganic Chemistry Laboratory, Department of Chemistry, University of Oxford, South Parks Road, Oxford OX1 3QR, UK.
2. Department of Materials, University of Oxford, Parks Road, Oxford, OX1 3PH, UK.
3. School of Chemical Engineering & Technology China University of Mining and Technology, Xuzhou, Jiangsu Province, 221116, China

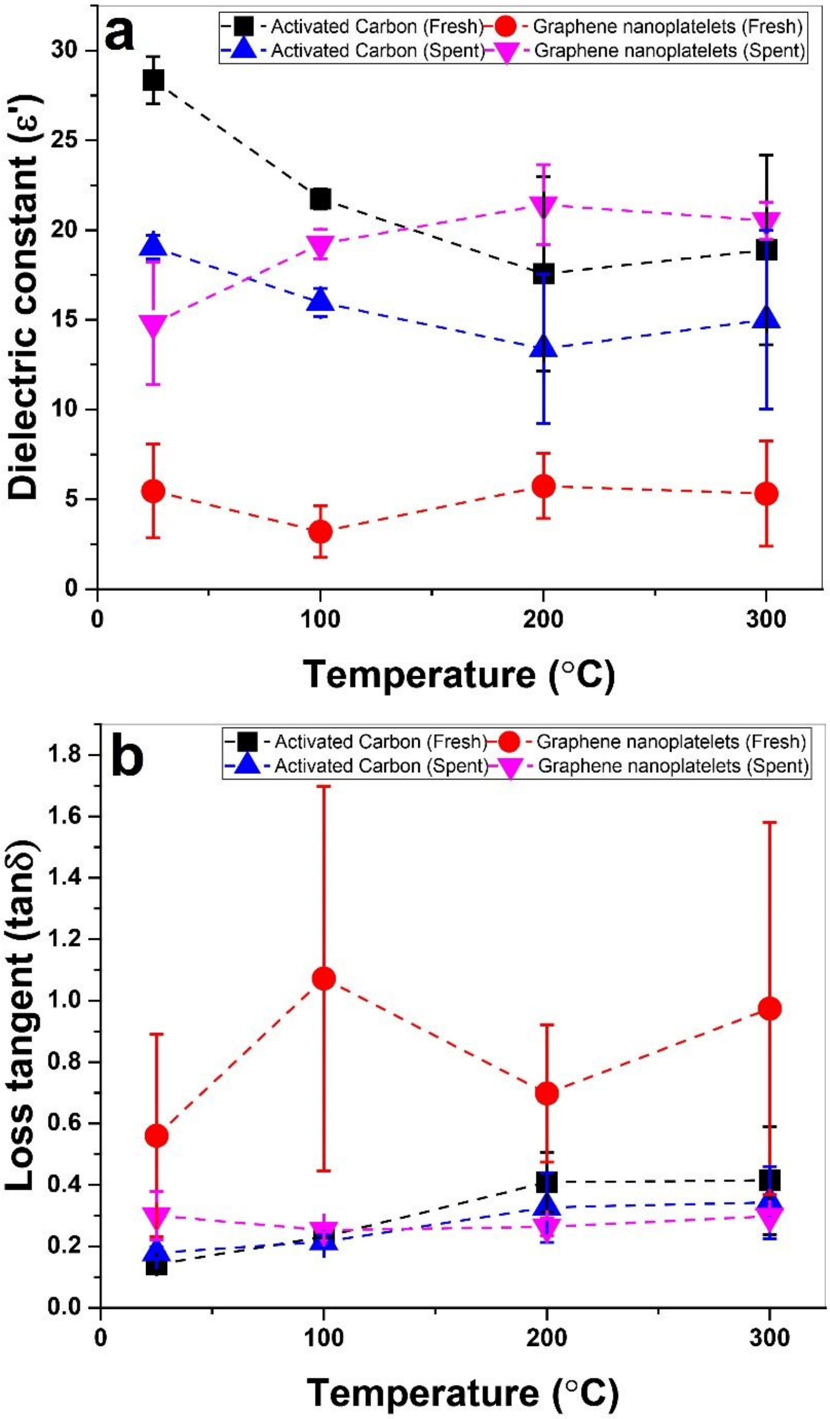

Figure S1. (a) Dielectric constant and (b) Loss tangent of activated carbon (ACs) and graphene nanoplatelets at different temperature, before and after catalytic reactions under microwave irradiation.

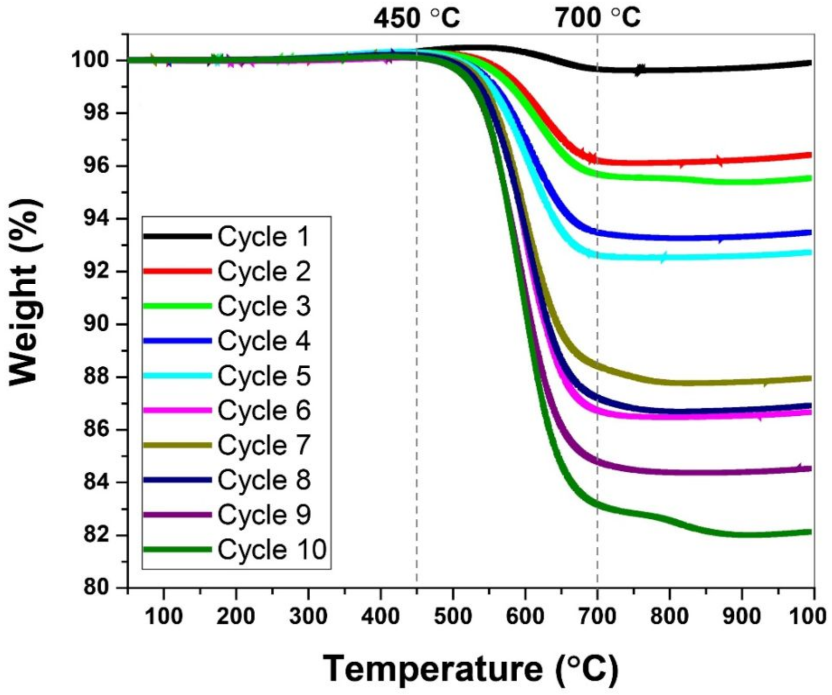

Figure S2. Thermogravimetric analysis (TGA).

Table S1. Oxidation temperatures of Model Carbon Materials and Fe/SiC sample.

Fe/SiC catalyst	Temperature (°C)	Model carbon Materials	Temperature (°C)
Cycle1	640	Activated carbon	653
Cycle2	625	Carbon black	745
Cycle3	619	Mesoporous Carbon	668
Cycle4	608	Graphene nanoplatelets	671
Cycle5	605	MWCNT	591
Cycle6	599	CNF	807
Cycle7	596	Graphite	853
Cycle8	603	Glassy carbon	808
Cycle9	596		
Cycle10	598		

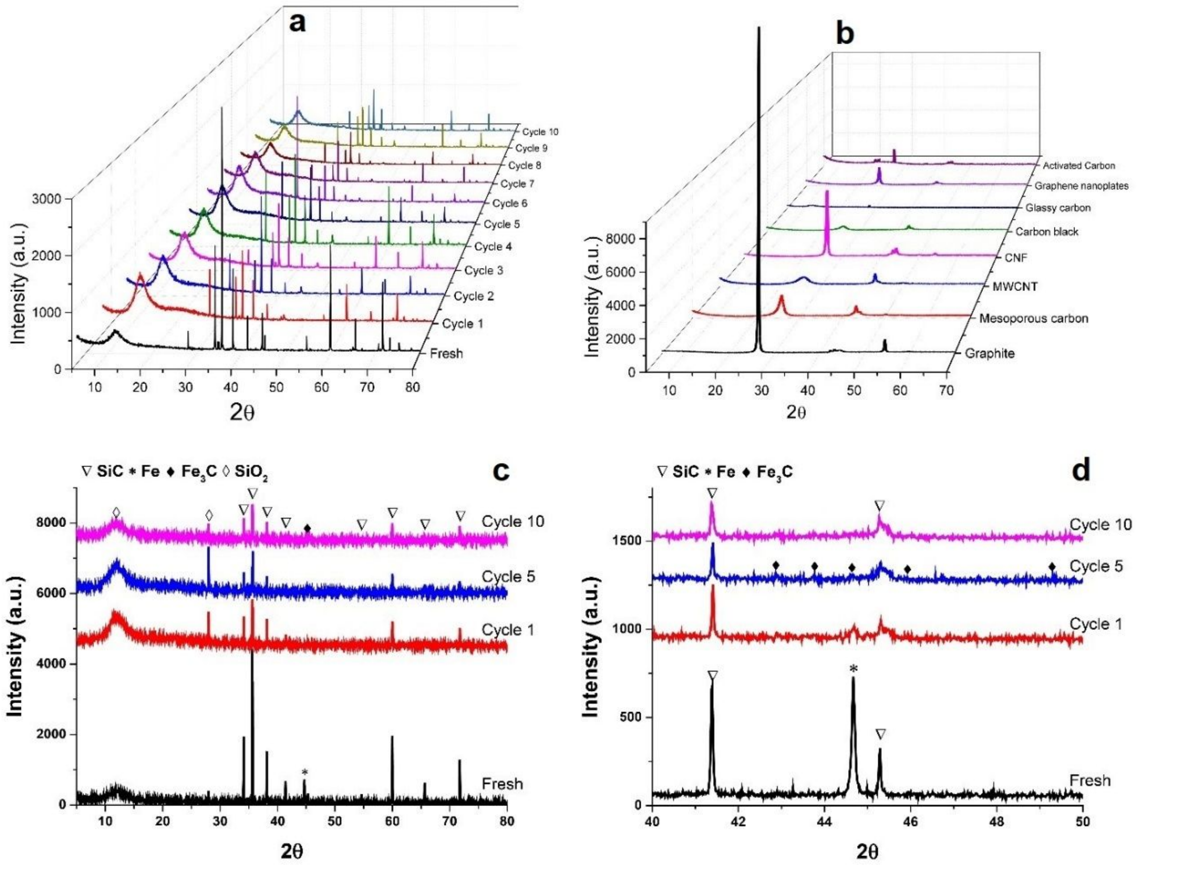

Figure S3. XRD patterns. (a) The change of the XRD pattern of 5 wt.% Fe/SiC sample during the successive cycles of hexadecane dehydrogenation under microwaves. (b) The XRD pattern of model carbon materials. (c) XRD pattern comparison of fresh Fe/SiC sample and after 1, 5, and 10 cycles, 2θ= 5°-80°, and (d) 2θ= 40°-50°.

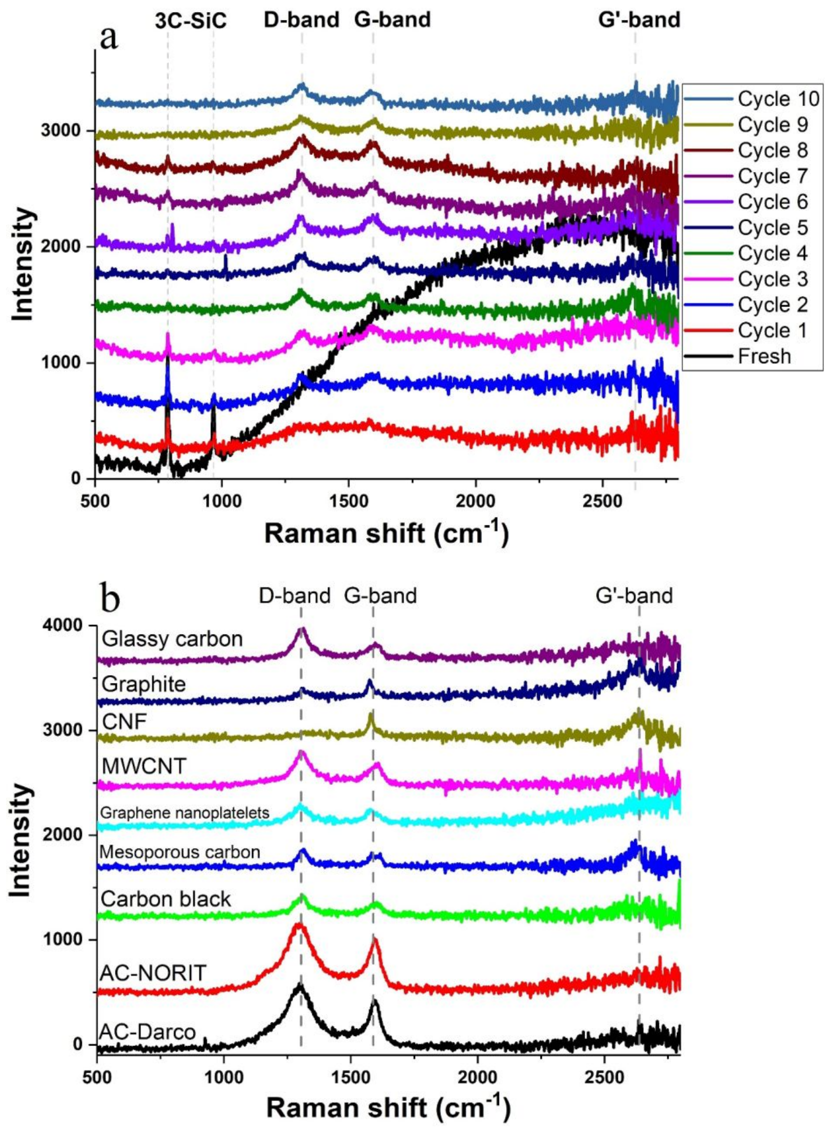

Figure S4. Raman Spectrum. (a) sample of Fe/SiC through 10 successive cycles of catalytic reaction. (b) sample of different model carbon materials.

Table S2. ID/IG of model carbon materials and samples collected at each cycle throughout the successive cycles of test

Model Carbons	ID/IG	Samples	ID/IG
AC-DARCO	1.39	Cycle 1	(-)
AC-NORIT	1.15	Cycle 2	0.99
CB	1.45	Cycle 3	0.97
MS	1.02	Cycle 4	1.03
GNPs	1.09	Cycle 5	1.01
MWCNT	1.04	Cycle 6	1.00
CNF	0.89	Cycle 7	1.04
Graphite	0.96	Cycle 8	1.03
GC	1.04	Cycle 9	1.01
		Cycle 10	1.02
